# Inferior Vena Cava Filter Use in the Setting of Gastrointestinal Blood Loss, Malignancy, and Multiple Thromboembolisms: A Case Report

**DOI:** 10.7759/cureus.28212

**Published:** 2022-08-20

**Authors:** Alexander Ahmann, Trent McElroy, Noah Stratton

**Affiliations:** 1 Anatomy, A.T. Still University, Kirksville College of Osteopathic Medicine, Kirksville, USA; 2 Internal Medicine, A.T. Still University, Kirksville College of Osteopathic Medicine, Kirksville, USA; 3 Internal Medicine, Baylor Scott & White Medical Center, Temple, USA

**Keywords:** primary colorectal cancer, retrievable inferior vena cava filter, deep vein thrombosis (dvt), malignancy-associated hypercoagulability, pulmonary embolism (pe)

## Abstract

Cancer-associated thromboembolism (CAT) is a common yet serious condition that occurs due to the physiological changes brought about by malignancy. The two conditions that are the most prevalent are deep vein thrombosis (DVT) and pulmonary embolism (PE). Anticoagulation is the standard of care for these thrombotic problems, however, in the event these medications are contraindicated, other treatment modalities may be needed. One common example is in the setting of an active bleed, such as gastrointestinal (GI) cancer. A treatment that has been used more frequently in recent years is the inferior vena cava (IVC) filter. These can be placed to provide a physical barrier to prevent a thrombus from moving through the circulation and potentially embolizing critical organs. An advantage of these devices is that they can be placed and removed when the use of pharmacological agents is better indicated. This report is a good example of a situation where an active GI malignancy created a hypercoagulable state leading to multiple thromboembolisms. An IVC filter was placed in the perioperative setting to prevent further thrombus migration while the primary malignancy was cured with a hemicolectomy.

## Introduction

Cancer-associated thromboembolism (CAT) is a common medical phenomenon; 20% of all thrombotic events occur in patients with neoplastic processes and they are the second leading cause of death in this population [1]. Deep vein thrombosis (DVT) is clotting that forms in the distal veins, and they have the potential to migrate to other locations which can have grave consequences. Pulmonary embolism (PE) is the most common and potentially life-threatening thromboembolism that is seen at a rate four times higher in patients with cancer than without [2]. Thrombotic events have an increased rate of incidence because of the body’s hypercoagulable state induced by neoplastic disease processes. Tissue factor (TF) and other cellular products are released into the circulation. They create inflammatory conditions and promote platelet adhesion as well as thrombin formation [3].

Treatment of CAT, in the setting of a DVT or PE is widely discussed in the literature, with chemical anticoagulation as the mainstay of treatment. Common pharmacologic agents include low-molecular-weight heparin (LMWH), warfarin, and more recently the use of direct oral anticoagulants [1]. The task of treating a CAT becomes more difficult in the setting of an active gastrointestinal (GI) bleed from malignancy. These lesions typically result in slow blood loss, which can lead to symptomatic anemia, and may require transfusions of packed red blood cells. In such cases, it may be inappropriate to use standard anticoagulation medication, as it can further exacerbate an active bleed.

Another treatment option available is the use of an inferior vena cava (IVC) filter. These devices are typically used in the perioperative setting where a patient is at high risk for thromboembolism, and when standard anticoagulation is contraindicated or must be stopped. In these scenarios, retrievable filters are generally used and should be removed once anticoagulation is started or restarted. Ideally, they should be removed within 30 days to decrease the risk of recurrent DVT or IVC filter thrombus formation [4]. This report highlights the appropriate use of a perioperative IVC filter in a 59-year-old African American male with a GI malignancy complicated by symptomatic anemia and multiple CATs.

## Case presentation

**Figure 1:**
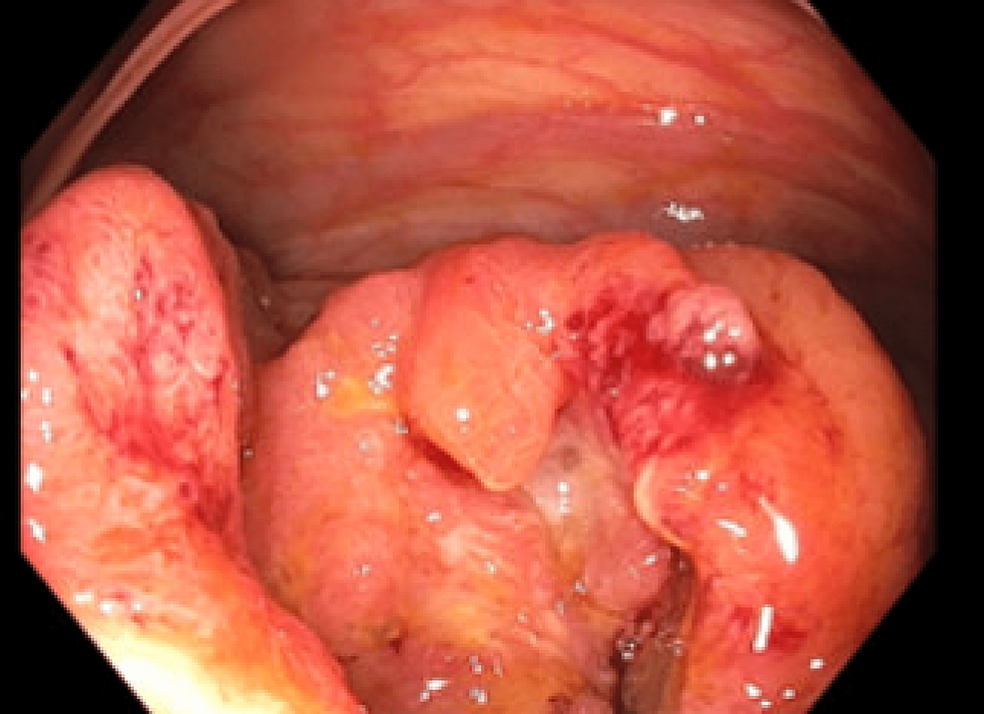
Cecal lesion discovered on diagnostic colonoscopy was confirmed as a stage IIA adenocarcinoma via biopsy

**Figure 2:**
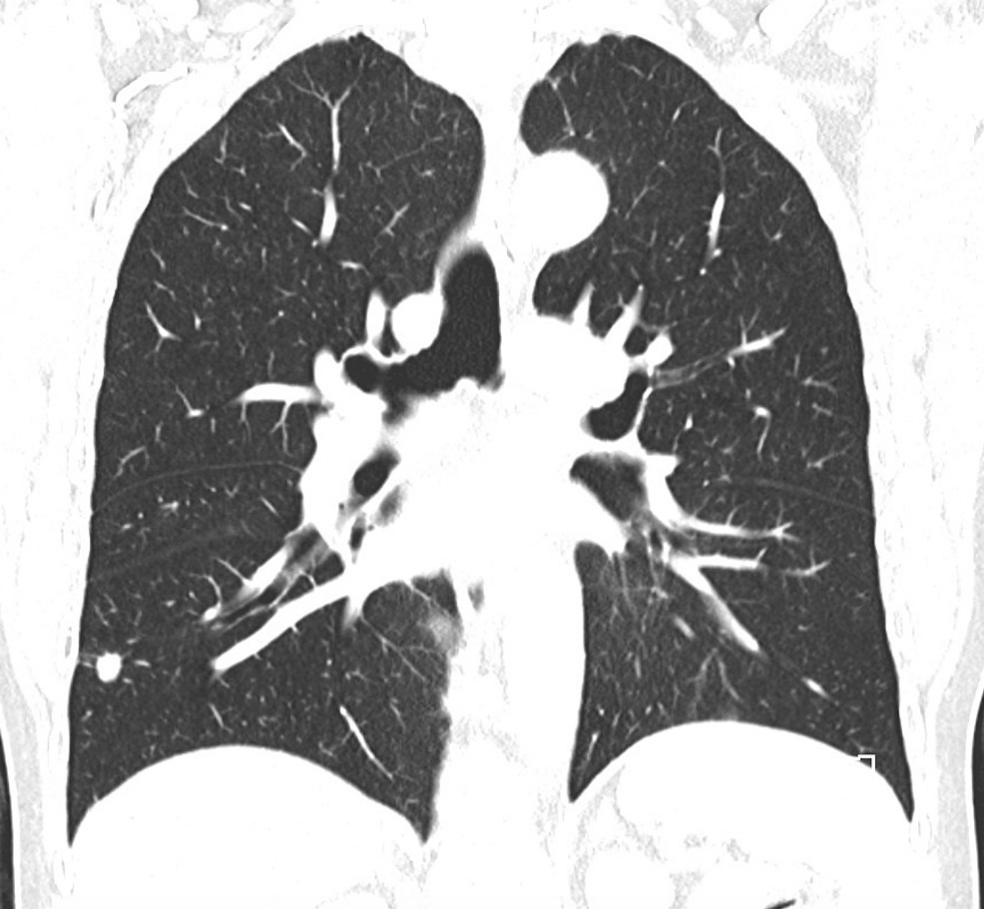
CT coronal section depicting right sided lung mass that was confirmed as a stage IA2 primary, epidermal growth factor receptor (EGFR) positive non-small cell lung cancer on biopsy

A 59-year-old African American male presented to the emergency department with a five-day history of right calf swelling and pain, as well as left calf pain. The patient has a significant medical history of hypertension, prediabetes, and iron deficient anemia, which resulted in hospitalization. Three months prior, he was diagnosed with a stage IIA adenocarcinoma of the cecum, and a stage IA2 primary, epidermal growth factor receptor (EGFR) positive non-small cell lung cancer of the right lower lobe (Figures 1-2). Before presenting to the emergency department, he was seen in a walk-in clinic with the current symptoms where bilateral lower extremity venous duplex ultrasound was conducted. Results of this exam demonstrated a right-sided, acute DVT from the mid femoral vein down through the distal right peroneal veins and a partially occlusive thrombus in the right posterior tibial veins. There was also a non-occlusive acute DVT on the left lower extremity extending from the left popliteal vein down through the distal posterior tibial and peroneal veins. Finally, it revealed superficial thrombophlebitis of the small saphenous vein. Upon discovery of these findings, the patient was transported to the emergency department for further evaluation and management. 

Initial vital signs in the emergency department showed a blood pressure of 151/94, pulse of 101, temperature of 100.2F, respiration rate of 19, and oxygen saturation of 99% on room air. Initial physical exam demonstrated swelling in the right lower extremity, tenderness to palpation bilaterally behind the knee, and the skin was warm to the touch. Bilateral digital clubbing was also noted. Review of symptoms was positive for a 20-pound weight loss in the last three months and was negative for chest pain, shortness of breath, dizziness, syncope, dark or bloody stools, bleeding, or craving ice. Initial lab studies were significant for hemoglobin of 5.8 and hematocrit of 21.5%; platelets were 470,000 and a stool guaiac test was positive.

Since his presenting conditions had a high suspicion of PE, a helical CT study was conducted. Results showed filling defects within the lobar branches of the right middle and lower lobes. Additional filling defects were identified within segmental and subsegmental branches of the right upper, middle, and lower lobes, as well as in the left lower lobe. Upon these findings in the emergency department, he was admitted to the internal medicine unit for further evaluation and management.

On the floor, he was given 2 units of packed red blood cells given the severe symptomatic anemia; he was started on a porcine heparin drip following hospital protocol at 20 units/kg/hr to treat the thromboembolisms. Hematology/oncology was consulted for further recommendations regarding anticoagulation in the setting of chronic slow lower GI bleed, and current bilateral DVTs, and PE. In addition, colorectal surgery was contacted because he was scheduled to undergo a right hemicolectomy the following day.

**Figure 3:**
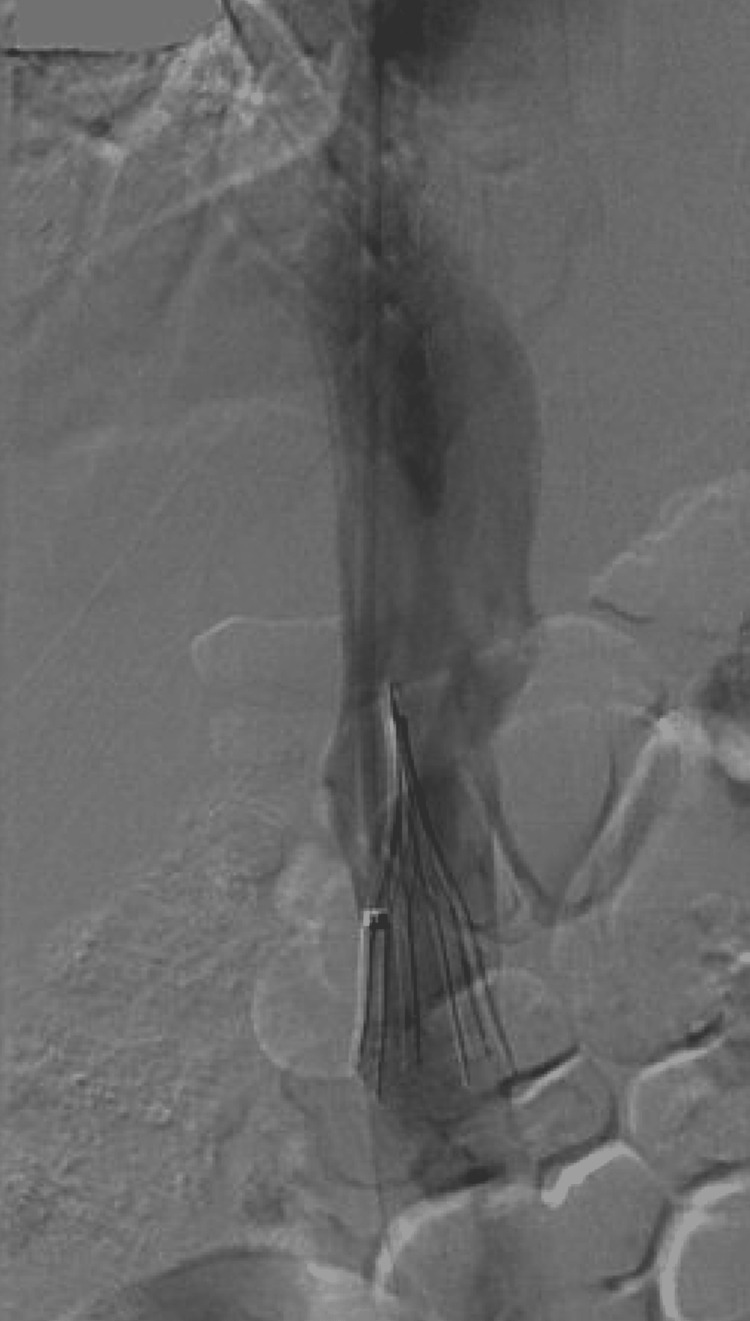
Fluoroscopic confirmation of inferior vena cava (IVC) filter deployment

Following transfusion, his hemoglobin levels increased to 8.2 and remained stable for the remainder of the hospital admission. Hematology and oncology recommended the placement of a retrievable IVC filter to be used in the acute perioperative setting. Colorectal surgery consultation resulted in the planned right hemicolectomy being postponed until successful placement of the IVC filter. Four days after admission, with the help of fluoroscopic and sonographic guidance, an Option Elite (Argon Medical Devices, Plano, TX, USA) IVC filter was placed infrarenal without complications (Figure 3).

On day seven of admission, the surgery team evaluated the patient and decided to move forward with the previously postponed procedure. They initiated bowel preparation and started the patient on a clear liquid diet which then transitioned to a 'no diet' at midnight with the maintenance of IV fluids prior to the operation. They also recommended pausing the porcine heparin drip for six hours leading up to surgery to avoid any excessive bleeding. The following day, a laparoscopic right hemicolectomy with intracorporeal ileocolonic anastomosis was successfully performed without any immediate postoperative complications. On the days following the procedure, the patient was able to eat a normal healthy diet and have bowel movements regularly and without pain or bleeding. One month following the initial hospitalization, he returned to have the IVC filter removed, which was accomplished without complication. At that time, he was followed by pulmonology and thoracic surgery to address plans for future procedures to address the lung adenocarcinoma.

## Discussion

CAT is an acute medical emergency superimposed on a chronic condition. While they are not uncommon, the sequelae can be devastating. This unique hypercoagulable environment is made possible due to an imbalance in hemostatic mechanisms that lead to an increase in thrombus formation and a decrease in lysis. The specific pathophysiological cause is complex and is induced when tumor cells express TF, pro-coagulant proteins, and metalloproteases. These products, in turn, activate endothelial cells, platelets, and leukocytes. Activated leukocytes produce and release other substances such as soluble and contact factors which lead to increased platelet adhesion and thrombin activation [3]. TF plays a role in the coagulation cascade by binding to factors VII and VIIa after injury to blood vessels. Significant levels of TF do not typically circulate in the blood without vascular injury, but various neoplastic processes are known to promote the expression of TF and may lead to a hypercoagulable state and promote thrombus formation [5].

GI malignancies are notorious for causing slow bleeds that can, in time, lead to symptomatic anemia due to blood loss. In many cases, this loss may not be obvious or apparent in the stool. Instead, there may be a slow and even microscopic blood loss that can lead to symptoms of fatigue, lightheadedness, and/or syncope over time. In such events when the hemoglobin level of the patient reaches a low enough level, below 7.0, transfusion of packed red blood cells may be necessary. In addition to the slow, chronic blood loss that can result from a GI malignancy, the risk of CAT is also present. 

Anticoagulation is the mainstay in treating CATs, however, in the setting of an active GI bleed, standard therapy might exacerbate an active bleed and make the symptoms of anemia worse. Common pharmacologic agents include the use of warfarin, heparin products, and direct oral anticoagulation. While these medications are effective in treating thromboembolisms, they come with an increased risk of bleeding. In the case presented, a porcine heparin drip was used to treat the CAT because it can be turned off quickly in the event of increased bleeding or for surgical interventions. However, to effectively prevent the recurrence of GI bleeding in these patients, the underlying malignancy must be treated. This accomplishes two important tasks. First, by treating the source of active blood loss, the patient’s anemia resolves naturally. Second, eliminating the malignant cells which caused the hypercoagulable state restores the natural physiology of hemostasis. In some situations, alternative treatments may need to be used as an adjunct to traditional methods. 

Current guidelines from the American Heart Association (AHA), American College of Chest Physicians (ACCP), and the American College of Radiology (ACR)/Society of Interventional Radiology (SIR) recommend the use of IVC filters in patients with venous thromboembolism who have an absolute contraindication to anticoagulation, in situations where pharmacologic intervention must be stopped, or have recurrent thrombus formation despite adequate anticoagulation [4]. The introduction of retrievable filters has slightly expanded the indication for use of IVC filters to include patients with large PE who are at risk for recurrent thrombus formation and embolization, those who are not compliant with medical management, patients with limited cardiopulmonary reserve, large free-floating DVT, patients with a high risk of complications from anticoagulation, such as active bleeding, and those who have CAT [4]. Even though the indications for use of an IVC filter have been expanded in recent years, the decision to use one is taken case by case, analyzing the risks and benefits of the intervention. 

The use of IVC filters is generally safe and well tolerated, however, nothing comes without risk. These risks are present during the initial placement of the filter, during the postoperative setting, and during retrieval. During the initial placement of the filters, the most common complication is due to bleeding and thrombosis formation. Less common issues can be due to improper placement, or migration, or it can be placed in the wrong location. These can lead to the filter not being able to operate properly or can lead to difficulty retrieving it when the indication is no longer present [4]. In the postoperative period, thrombosis is also possible, leading to an increased risk of developing a DVT, PE, or even renal failure if a thrombus affects the renal vasculature. Perforation of the IVC is also possible because hooks present on the filters to prevent migration may injure vessels. Filters are made of strong metallic materials; however, they can fail and send shards up the circulation to various vital organs such as the heart and lungs [2]. Finally, serious complications exist at the time of filter retrieval. During this period, it is possible to create an injury or dissection to the IVC or have a failure of the filter causing fragmentation. It is generally recommended that the filters are removed when the increased risks of CAT or another thrombus formation have passed. This is generally done within 30 days of initial implantation [4].

## Conclusions

The art of medicine is present in a case like this because one must weigh the risks and benefits of treatment modalities when multiple conflicting problems exist. The provider must decide which problem is the most acute and attack that problem first before less acute issues can be treated. In complex disease processes like the one presented, one must take a holistic approach and make informed decisions that will benefit the patient the most. There is no standard treatment for each person because every patient and situation is different from the next. Even though there may be similarities between cases, each case is ultimately unique and may require different treatments for the disease processes involved.
